# Changes in Distal Tibial Microarchitecture During Eight Weeks of U.S. Army Basic Combat Training Differ by Sex and Race

**DOI:** 10.1002/jbm4.10719

**Published:** 2023-03-02

**Authors:** Julie M. Hughes, Kathryn M. Taylor, Katelyn I. Guerriere, Nathaniel I. Smith, Jeffery S. Staab, Leila A. Walker, Janet E. Staab, Paul M. Bartlett, Barry A. Spiering, Vy T. Nguyen, Susan P. Proctor, Stephen A. Foulis, Mary L. Bouxsein, Kristin L. Popp

**Affiliations:** ^1^ Military Performance Division United States Army Research Institute of Environmental Medicine Natick MA USA; ^2^ Research Service VA Boston Healthcare System Boston MA USA; ^3^ Endocrine Unit Massachusetts General Hospital Boston MA USA; ^4^ Center for Advanced Orthopedic Studies Beth Israel Deaconess Medical Center Boston MA USA; ^5^ Department of Orthopedic Surgery Harvard Medical School Boston MA USA

**Keywords:** BONE FORMATION, EXERCISE, HR‐PQCT, MILITARY TRAINING

## Abstract

Basic combat training (BCT) is a physically rigorous period at the beginning of a soldier's career that induces bone formation in the tibia. Race and sex are determinants of bone properties in young adults but their influences on changes in bone microarchitecture during BCT are unknown. The purpose of this work was to determine the influence of sex and race on changes in bone microarchitecture during BCT. Bone microarchitecture was assessed at the distal tibia via high‐resolution peripheral quantitative computed tomography at the beginning and end of 8 weeks of BCT in a multiracial cohort of trainees (552 female, 1053 male; mean ± standard deviation [SD] age = 20.7 ± 3.7 years) of which 25.4% self‐identified as black, 19.5% as race other than black or white (other races combined), and 55.1% as white. We used linear regression models to determine whether changes in bone microarchitecture due to BCT differed by race or sex, after adjusting for age, height, weight, physical activity, and tobacco use. We found that trabecular bone density (Tb.BMD), thickness (Tb.Th), and volume (Tb.BV/TV), as well as cortical BMD (Ct.BMD) and thickness (Ct.Th) increased following BCT in both sexes and across racial groups (+0.32% to +1.87%, all *p* < 0.01). Compared to males, females had greater increases in Tb.BMD (+1.87% versus +1.40%; *p* = 0.01) and Tb.Th (+0.87% versus +0.58%; *p* = 0.02), but smaller increases in Ct.BMD (+0.35% versus +0.61%; *p* < 0.01). Compared to black trainees, white trainees had greater increases in Tb.Th (+0.82% versus +0.61%; *p* = 0.03). Other races combined and white trainees had greater increases in Ct.BMD than black trainees (+0.56% and + 0.55% versus +0.32%; both *p* ≤ 0.01). Changes in distal tibial microarchitecture, consistent with adaptive bone formation, occur in trainees of all races and sexes, with modest differences by sex and race. Published 2023. This article is a U.S. Government work and is in the public domain in the USA. *JBMR Plus* published by Wiley Periodicals LLC on behalf of American Society for Bone and Mineral Research.

## Introduction

Skeletal adaptation to initial military training is thought to be a key factor in reducing the incidence of lower extremity stress fractures, which are one of the most common injuries associated with military training.^(^ ^1^ ^)^ The physical activity that occurs during basic combat training (BCT), a rigorous period at the beginning of a soldier's career, causes adaptive bone formation in the tibia, which is a common location for stress fractures.^(^ ^2^, ^3^, ^4^, ^5^ ^)^ Adaptive bone formation may confer increases in bone strength^(^ ^6^ ^)^ and fatigue resistance,^(^ ^7^ ^)^ which could have important implications for stress fracture prevention.^(^ ^8^ ^)^

During military training, females have a threefold greater risk of stress fractures than males.^(^ ^8^, ^9^ ^)^ However, the prevalence of stress fracture is greater in males because there are more males than females in BCT.^(^ ^10^ ^)^ Therefore, skeletal adaptations to BCT are relevant for both males and females. The greater rates of stress fracture in females than males may be due in part to sex‐based differences in bone properties at the start of BCT and/or sex‐based differences in the skeletal response to BCT. On average, females have less favorable bone properties at the start of BCT than males.^(^ ^11^, ^12^, ^13^ ^)^ Sex‐specific differences in bone density, macroarchitecture, and microarchitecture at the start of BCT may influence the relative magnitude of bone strain experienced during training, which may in turn lead to differences in adaptive bone formation in response to BCT. In alignment with this concept, volumetric bone density at the distal tibia increased in both males and females following 14 weeks of British Army basic training, but increases were greater in females than males.^(^ ^14^ ^)^ Whether changes in bone microarchitecture that are indicative of adaptive bone formation differ in females and males during military training remains to be determined.

Stress fracture incidence also varies by race and ethnic background,^(^ ^9^, ^15^ ^)^ with black soldiers experiencing the lowest risk, white soldiers the highest risk, and Asian and Hispanic soldiers at intermediate risk.^(^ ^8^ ^)^ Bone density and strength varies by race in young adults, with black males and females generally having higher bone density and more favorable bone microarchitecture compared to white males and females.^(^ ^12^, ^13^ ^)^ These differences in bone properties may contribute to differences in stress fracture risk, but disparities in bone properties at the start of BCT may also influence bone adaptations to BCT.

To better understand the skeletal response to initial military training and how it might differ by sex and race, we conducted a longitudinal observational study of bone microarchitecture changes during BCT in a multiracial cohort of males and females. We hypothesized that BCT would lead to favorable changes in tibial bone density, macroarchitecture, and microarchitecture, consistent with adaptive bone formation, in both males and females, irrespective of race. We further hypothesized that males and black trainees would exhibit more favorable bone microarchitecture prior to BCT than females and trainees who self‐identify as a race other than black, but that these latter groups will exhibit relatively greater changes in bone microarchitecture, consistent with adaptive bone formation, than males and black trainees during BCT.

## Subjects and Methods

### Participants

Male and female U.S. Army trainees between 17 and 42 years of age were recruited from Fort Jackson, SC, USA and Fort Sill, OK, USA for this prospective observational study. Volunteer eligibility and enrollment have been described.^(^ ^16^ ^)^ In brief, trainees were eligible to participate if they were not pregnant or breastfeeding, not restricted from physical activity by a medical provider or otherwise suffering from an injury that limited exercise, and did not have history of an endocrine disorder, bone‐modifying disorder, or glucocorticoid drug prescription within 2 years of the study. Prior to enrollment, trainees were briefed on the voluntary nature of this study without command staff present and were given time to consider their participation and ask questions of an ombudsperson. Participants provided signed informed written consent for research approved by the Institutional Review Board of the U.S. Army Medical Research & Development Command, Fort Detrick, MD, USA. Per Department of Defense Instruction (DoDI) 3216.02, trainees who are 17 years of age were considered adults while in federal duty status and were allowed to consent without parent or guardian approval. The upper age limit for inclusion criteria of 42 years was selected because it corresponds with the upper age limit for enlistment, without a waiver, into the U.S. Army.

**Fig. 1:**
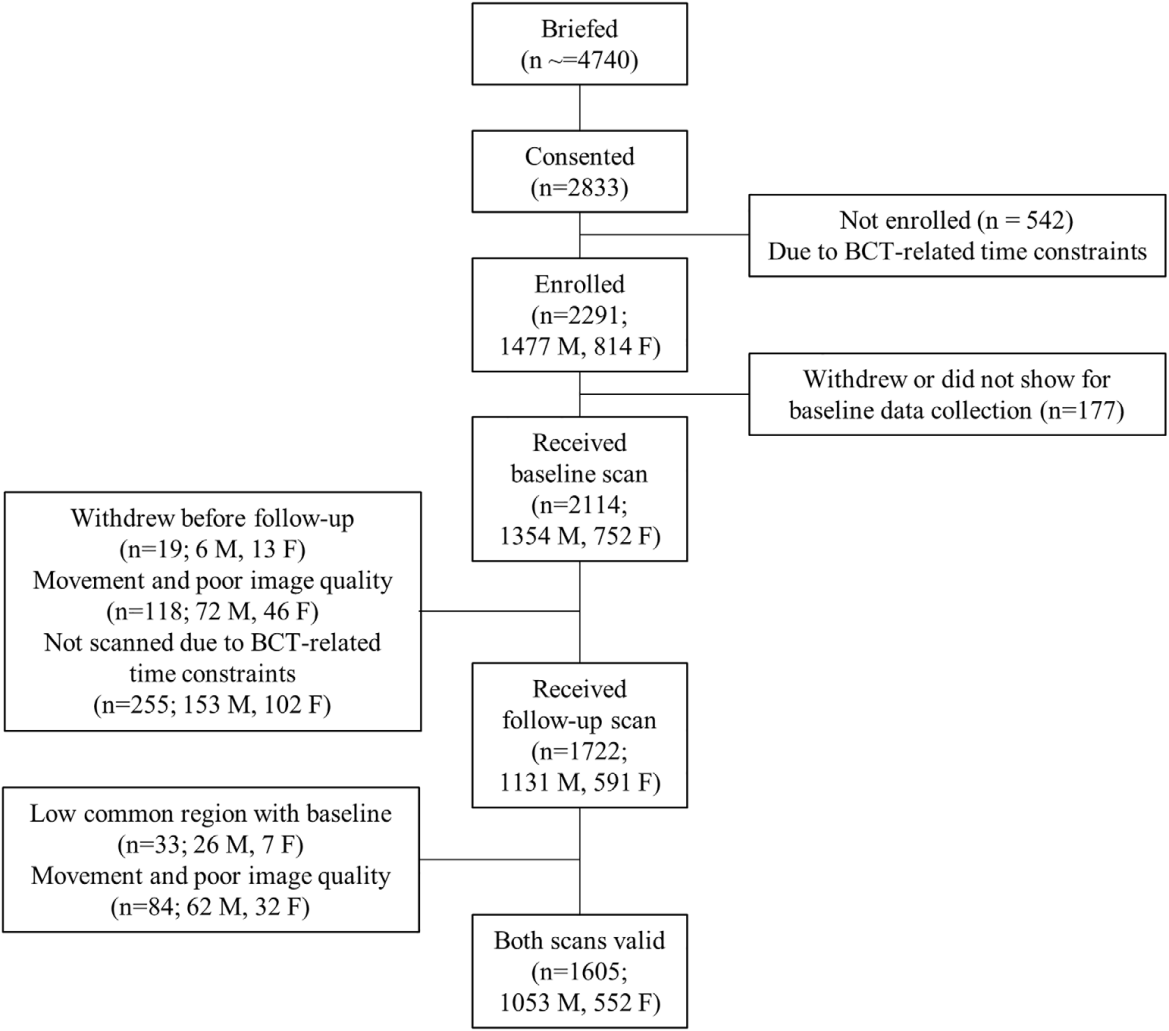
Consort diagram.

Approximately 4749 trainees were briefed for the study between August 2017 and October 2019, of which 2291 were enrolled and 1605 were included in the present analysis (Fig. 1). The most common explanations for volunteer dropout included not wanting to miss training, reporting to sick call, unspecified voluntary withdrawal, being discharged or recycled (begin training again in a future training cycle), and logistical conflicts with the BCT schedule.

Volunteers completed baseline data collection during the first week of BCT, and follow‐up data collection 8 weeks later, as described.^(^ ^16^ ^)^ Activities that comprise BCT were not altered due to participation in this observational study. As BCT dictates, volunteers were provided three meals per day in an on‐site dining facility, and underwent military specific training including road marches with load carriage, running, resistance and calisthenics exercises such as push‐ups and sit‐ups, hand‐to‐hand combat, and weapons training.^(^ ^9^, ^17^ ^)^

### Background and health status surveys

Volunteers completed questionnaires at baseline as described.^(^ ^16^ ^)^ Briefly, volunteers provided information pertaining to their demographics, such as age, race (Asian, black, Indian or Indian Subcontinent, Native American/Alaskan Native, Native Hawaiian/Pacific Islander, white) and sex (female, male). Due to modest sample sizes for Asian, Indian or Indian Subcontinent, Native American/Alaskan Native, and Native Hawaiian/Pacific Islander trainees, those within these races were aggregated into a category termed Other Races Combined (ORC).

Additional questionnaires gathered information related to lifestyle (eg, smoking history) and physical activity, both of which are known to influence bone properties. Self‐reported smoking history was categorized as ever (y/n) or current (y/n; last use of tobacco product was within 30 days of BCT start date). Self‐reported physical activity was categorized as having participated in 30 or more minutes of physical activity in the 60 days prior to BCT for 0–2 days/week, 3–4 days/week, or 5 or more days/week. Females also reported their current use of hormonal contraceptives. Questionnaires were self‐administered under the supervision of study staff.

### Anthropometrics

Standing height was measured to the nearest centimeter at baseline using a stadiometer (Creative Health Products, Plymouth, MI, USA). Body mass was measured to the nearest 0.1 kg at pre‐BCT and post‐BCT using a calibrated digital scale (Doran Scales, St. Charles, IL, USA; model DS6150). Height and body mass measurements were collected with volunteers dressed in their physical training shirt and shorts or similar attire, without shoes.

### High‐resolution peripheral quantitative computed tomography

We acquired high‐resolution peripheral quantitative computed tomography (HR‐pQCT) (XTremeCT II; Scanco Medical AG, Brüttisellen, Switzerland) scans to assess morphologic and microarchitectural parameters at the distal metaphyseal region of the tibia, as described.^(^ ^16^ ^)^ In brief, HR‐pQCT scans were obtained at baseline and follow‐up from the nondominant leg, unless the participant reported a prior fracture in that leg, in which case the dominant leg was scanned. Leg dominance was self‐selected by trainees as the preferred leg for kicking a ball. Trained study personnel measured tibia length from the distal aspect of the medial malleolus to the medial border of the tibial plateau to the nearest 0.5 cm using a flexible tape measure, and scans were conducted at 4% of the tibia length from the distal endplate. The baseline tibia length measurement was used at follow‐up, in accordance with best practices for longitudinal studies.^(^ ^18^ ^)^ All scans were reviewed immediately for movement artifact which was scored on a five‐point scale, with one equivalent to no movement and five equivalent to severe movement artifact.^(^ ^19^ ^)^ Scans were repeated up to two times if movement artifact was graded four or five. Quality control scans were completed daily using the manufacturer's calibration phantom, which contains five regions of hydroxyapatite‐resin mixtures of known mineral density (Scanco Medical AG).

All HR‐pQCT processing and analyses were conducted using manufacturer‐provided software (Image Processing Language, v5.42; Scanco Medical AG) as described.^(^ ^20^, ^21^ ^)^ Bone mineral density (BMD) values for the cortical and trabecular compartments (Ct.BMD and Tb.BMD, respectively) as well as for the entire region (Tt.BMD) were calculated as the mean mineralization value within each region, using the density calibration. Cortical thickness (Ct.Th) was obtained from the three‐dimensional (3D) reconstructed volumes and represents a direct measure of the average periosteal‐endosteal distance. Mineralized bone was distinguished from marrow space in the trabecular compartment using a low‐pass Gaussian filter and a fixed density threshold of 320 mg hydroxyapatite (HA)/cm^3^. Trabecular bone volume (Tb.BV/TV) was calculated as the ratio of mineralized voxels to total voxels within the trabecular compartment. Trabecular number (Tb.N), thickness (Tb.Th), and separation (Tb.Sp) were each measured directly using 3D methods. Short‐term reproducibility with repositioning (mean coefficients of variation) for HR‐pQCT measurements at the 4% tibia site in our laboratory are 0.44% to 0.61% for density parameters, 0.68% to 1.55% for microarchitecture parameters, and 1.85% for Ct.Th. The common volume between paired baseline and follow‐up scans for each volunteer was determined using the manufacturer's default cross‐sectional area‐based registration scheme (Image Processing Language, v5.42, Scanco Medical AG). This method corrects for differences in axial position of the limb within the scanner between baseline and follow‐up scans. Only the bone volume common to both scans of a given pair was included in these analyses, and scan pairs for which the common volume was less than 85% (*n* = 33) were excluded. In total, 118 baseline scans and 117 follow‐up scans were excluded due to movement artifact, poor image quality, or low common region (Fig. 1). We calculated the within‐scanner least‐significant change (LSC), as 2.77 times the precision error, to determine the least amount of change in Tt.BMD and Tb.BMD, within an individual participant that can be considered statistically significant.

### Power calculation

We estimated that a sample size of 1467 individuals was needed to have sufficient power (>80%) to detect a difference (alpha < 0.05) in changes in bone variables among males and females and trainees of black, white, and ORC. This sample size estimate was calculated using repeated study simulations within a simulated population that assumed similar distributions of race, sex, and each of the bone microarchitectural parameters based on a prior study in BCT.^(^ ^2^ ^)^ Specifically, we assumed between 0.34% and 2.01% change (versus baseline) in each of the bone parameters, as well as a percent difference between groups as low as 0.20%. We assumed the breakdown of race was 25% black, 20% ORC, 55% white, and the breakdown for sex was 34% females and 66% males. Monte Carlo methods were used to randomly sample individuals from simulated populations to create 1000 pseudo study populations. Within these populations, proposed models were evaluated and the proportion of simulated studies that had a *p* value < 0.05 for the relationships of interest was equal to the study power.

### Statistical analyses

We computed descriptive statistics for the enrolled population using frequencies and percent of population for categorical variables and means and standard deviations (SDs) for the continuous variables. To account for the small amount of missing data in reporting of race, multiple imputation with five iterations was used. Linear regression models were used to estimate the adjusted mean differences and their corresponding 95% confidence intervals (CIs) in baseline values as well as mean percent change in each microarchitectural bone measurements comparing males and females, and trainees of black, white, and ORC. Pairwise comparisons were used to compare model coefficients between the three race groups in order to assess statistical significance. For each of the bone microarchitectural measurements, we ran a model for the percent change from baseline for each participant. We also repeated the analyses using the absolute change in each bone variable. All models were adjusted for sex when comparing bone changes by race and for race when comparing bone changes by sex. Additional model covariates included age, height, weight, current tobacco use, and prior physical activity. In addition, we examined whether it was necessary to adjust models in females for self‐reported hormonal contraceptive use. Final models were determined using Akaike's information criterion (AIC) to determine the model with the best goodness of fit. Analyses were conducted with SAS statistical software (v 9.4; SAS Institute, Cary, NC, USA).

## Results

### Participant characteristics

We enrolled 2291 trainees, of whom 1605 participants (1053 males and 552 females) had acceptable baseline and follow‐up HR‐pQCT scans (Fig. 1, Table 1). On average (mean ± SD), females and males were young (20.7 ± 3.7 years), and their body mass index (BMI) was 24.7 ± 3.5 kg/m^2^. The cohort was comprised of 398 black (25.4%) and 865 white (55.1%) individuals, with 306 individuals classified as ORC (19.5%). The ORC group included individuals who identified as Asian (*n* = 77), Indian or from an Indian subcontinent (*n* = 4), Native American/Alaskan American (*n* = 25), Native Hawaiian/Pacific Islander (*n* = 15), other (*n* = 91), or mixed race (*n* = 94). Of the 1605 participants included in the analyses, 19 (1.18%) had a history of stress fracture prior to BCT (six females, 12 males; two black, two ORC, and 15 white trainees). Of the 552 females in the study, 143 (25.9%) reported current use of hormonal contraceptives at baseline, 406 (73.5%) reported no current use, and three (0.5%) did not answer this question on the survey. Self‐reported current hormonal contraceptive use had minimal influence on skeletal response to BCT (<10% change in the adjusted mean estimates), thus it was not included as a covariate in final statistical models among females.

**Table 1:** Demographics and Group Characteristics for the Total Cohort of Trainees by Sex and Race

Baseline characteristics	Total (*n* = 1605)	Females (*n* = 552)	Males (*n* = 1053)	Black (*n* = 398)	ORC (*n* = 306)	White (*n* = 865)
Age (years), mean ± SD	20.7 ± 3.7	20.3 ± 3.5	21.0 ± 3.7	20.6 ± 3.8	21.3 ± 4.1	20.6 ± 3.5
Race, *n* (%)						
Black	398 (24.8)	174 (31.5)	224 (21.3)			
ORC	306 (19.1)	119 (21.6)	187 (17.7)			
White	865 (53.9)	248 (44.9)	617 (58.6)			
Missing	36 (2.2)	11(2.0)	25(2.4)			
Sex (% Female), *n* (%)				174 (32.2)	119 (22.0)	248 (45.8)
Height (cm), mean ± SD	170.9 ± 9.6	161.7 ± 6.3	175.8 ± 7.2	170.4 ± 9.0	167.9 ± 10.2	172.4 ± 9.4
Weight (kg), mean ± SD	72.3 ± 13.6	62.8 ± 8.7	77.4 ± 13.0	72.3 ± 13.8	70.2 ± 14.2	73.1 ± 13.2
Physical activity, *n* (%)						
Low, 0–2 day/week	312 (19.4)	130 (23.6)	182 (17.3)	102 (25.6)	53 (17.3)	151 (17.5)
Moderate, 3–4 day/week	597 (37.2)	229 (41.5)	368 (35.0)	153 (38.4)	122 (39.9)	306 (35.4)
High, 5+ day/week	696 (43.4)	193 (35.0)	503 (47.8)	143 (35.9)	131 (42.8)	408 (47.2)
Smoking, *n* (%)						
Ever (yes/no)	328 (20.4)	60 (10.9)	268 (25.5)	53 (13.3)	52 (17.0)	220 (25.4)
Current (yes/no)	216 (13.5)	32 (5.8)	184 (17.5)	24 (6.0)	30 (9.8)	160 (18.5)

Abbreviation: ORC = other races combined.

### Baseline bone density and microarchitecture at the distal tibia

At BCT entry, males had more favorable bone properties than females, even after adjusting for body weight and other covariates, including greater cortical area (Ct.Ar), Tt.BMD, Tb.BMD, Tb.BV/TV, Tb.Th, Tb.N, Ct.Th (all *p* < 0.05) and lower Tb.Sp and Ct.BMD (*p* < 0.01; Table 2). Black and ORC trainees had greater Ct.Ar, Tt.BMD, Tb.BMD, Tb.BV/TV, Tb.Th, Tb.Sp, Ct.BMD, and Ct.Th, and lower Tb.N than white trainees even after adjusting for body weight and other covariates (all *p* ≤ 0.01; Table 3). Black trainees also had greater Tt.BMD, Tb.BMD, Tb.BV/TV, Tb.Th, Ct.BMD, Ct.Ar, and Ct.Th (all *p* < 0.01) than those characterized as ORC even after adjustment for body weight and other covariates. All unadjusted results between sexes and races are presented in Tables S1 and S2, respectively.

**Table 2:** Pre‐Basic Combat Training Values for Bone Parameters in Males and Females Adjusted for Age, Height, Weight, Race, Physical Activity, and Tobacco Use

Variable	Males (*n* = 1053) mean (95% CI)	Females (*n* = 552) mean (95% CI)	Males versus females	
			Mean difference (95% CI)	*p*
Tt.BMD (mg HA/cm^3^)	278 (275, 281)	253 (248, 257)	25 (20, 31)	<0.001
Tb.BMD (mg HA/cm^3^)	223 (221, 225)	199 (196, 203)	24(19, 28)	<0.001
Tb.BV/TV (%)	33.0 (32.7, 33.4)	29.4 (28.9, 30.0)	3.6 (2.9, 4.3)	<0.001
Tb.Th (mm)	0.247 (0.246, 0.249)	0.232 (0.230, 0.234)	0.015 (0.013, 0.018)	<0.001
Tb.N (1/mm)	1.77 (1.75, 1.78)	1.74 (1.71, 1.76)	0.03 (0.00, 0.06)	0.040
Tb.Sp (mm)	0.522 (0.517, 0.528)	0.538 (0.530, 0.546)	−0.016 (−0.026, −0.005)	0.003
Ct.BMD (mg HA/cm^3^)	801 (798, 804)	828 (823, 832)	−27 (−33, −21)	<0.001
Ct.Ar (mm^2^)	107 (106, 109)	89 (87, 91)	18 (15, 21)	<0.001
Ct.Th (mm)	0.937 (0.922, 0.951)	0.805 (0.784, 0.826)	0.132 (0.104, 0.159)	<0.001

Abbreviation: Ct.Ar = cortical area; Ct.BMD = cortical volumetric bone mineral density; Ct.Th = cortical thickness; Tb.BMD = trabecular volumetric bone mineral density; Tb.BV/TV = trabecular bone volume/total volume; Tb.N = trabecular number; Tb.Sp = trabecular separation; Tb.Th = trabecular thickness; Tt.BMD = total volumetric bone mineral density.

**Table 3:** Pre‐Basic Combat Training Values for Bone Parameters in Trainees of Black, ORC, and White Race Adjusted for Age, Height, Weight, Sex, Physical Activity, and Tobacco Use

				Black versus ORC		Black versus white		ORC versus white	
Variable	Black (*n* = 398) mean (95% CI)	ORC (*n* = 306) mean (95% CI)	White (*n* = 865) mean (95% CI)	Mean difference (95% CI)	*p*	Mean difference (95% CI)	*p*	Mean difference (95% CI)	*p*
Tt.BMD (mg HA/cm^3^)	280 (276, 284)	263 (258, 267)	253 (250, 256)	17 (12, 23)	<0.001	27 (23, 32)	<0.001	10 (5, 15)	<0.001
Tb.BMD (mg HA/cm^3^)	219 (216, 222)	210 (206, 214)	204 (202, 207)	9 (4, 14)	<0.001	15 (11, 19)	<0.001	6 (1, 10)	0.011
Tb.BV/TV (%)	32.5 (32.1, 33.0)	31.1 (30.5, 31.6)	30.1 (29.8, 30.5)	1.5 (0.7, 2.2)	<0.001	2.4 (1.8, 3.0)	<0.001	0.9 (0.3, 1.6)	0.003
Tb.Th (mm)	0.247 (0.245, 0.248)	0.239 (0.237, 0.241)	0.234 (0.232, 0.235)	0.008 (0.005, 0.010)	<0.001	0.013 (0.011, 0.015)	<0.001	0.005 (0.003, 0.008)	<0.001
Tb.N (1/mm)	1.72 (1.70, 1.74)	1.75 (1.72, 1.77)	1.79 (1.77, 1.80)	−0.03 (−0.06, 0.00)	0.069	−0.07 (−0.09, −0.04)	<0.001	−0.04 (−0.07, −0.01)	0.003
Tb.Sp (mm)	0.537 (0.530, 0.545)	0.533 (0.524, 0.541)	0.521 (0.515, 0.526)	0.005 (−0.006, 0.016)	0.375	0.017 (0.008, 0.026)	<0.001	0.012 (0.002, 0.021)	0.015
Ct.BMD (mg HA/cm^3^)	836 (832, 840)	808 (803, 813)	798 (795, 801)	27 (21, 34)	<0.001	37 (32, 42)	<0.001	10 (4, 15)	<0.001
Ct.Ar (mm^2^)	107 (106, 109)	97 (95, 100)	90 (89, 92)	10 (7, 13)	<0.001	17 (15, 19)	<0.001	7 (5, 10)	<0.001
Ct.Th (mm)	0.964 (0.946, 0.983)	0.859 (0.837, 0.882)	0.789 (0.775, 0.803)	0.105 (0.077, 0.134)	<0.001	0.176 (0.153, 0.198)	<0.001	0.070 (0.045, 0.095)	<0.001

Abbreviation: Ct.Ar = cortical area; Ct.Th = cortical thickness; Ct.BMD = cortical volumetric bone mineral density; ORC = other races combined; Tb.BMD = trabecular volumetric bone mineral density; Tb.BV/TV = trabecular bone volume/total volume; Tb.N = trabecular number; Tb.Sp = trabecular separation; Tb.Th = trabecular thickness; Tt.BMD = total volumetric bone mineral density.

### Changes in bone density and microstructure during BCT: influence of sex

**Fig. 2:**
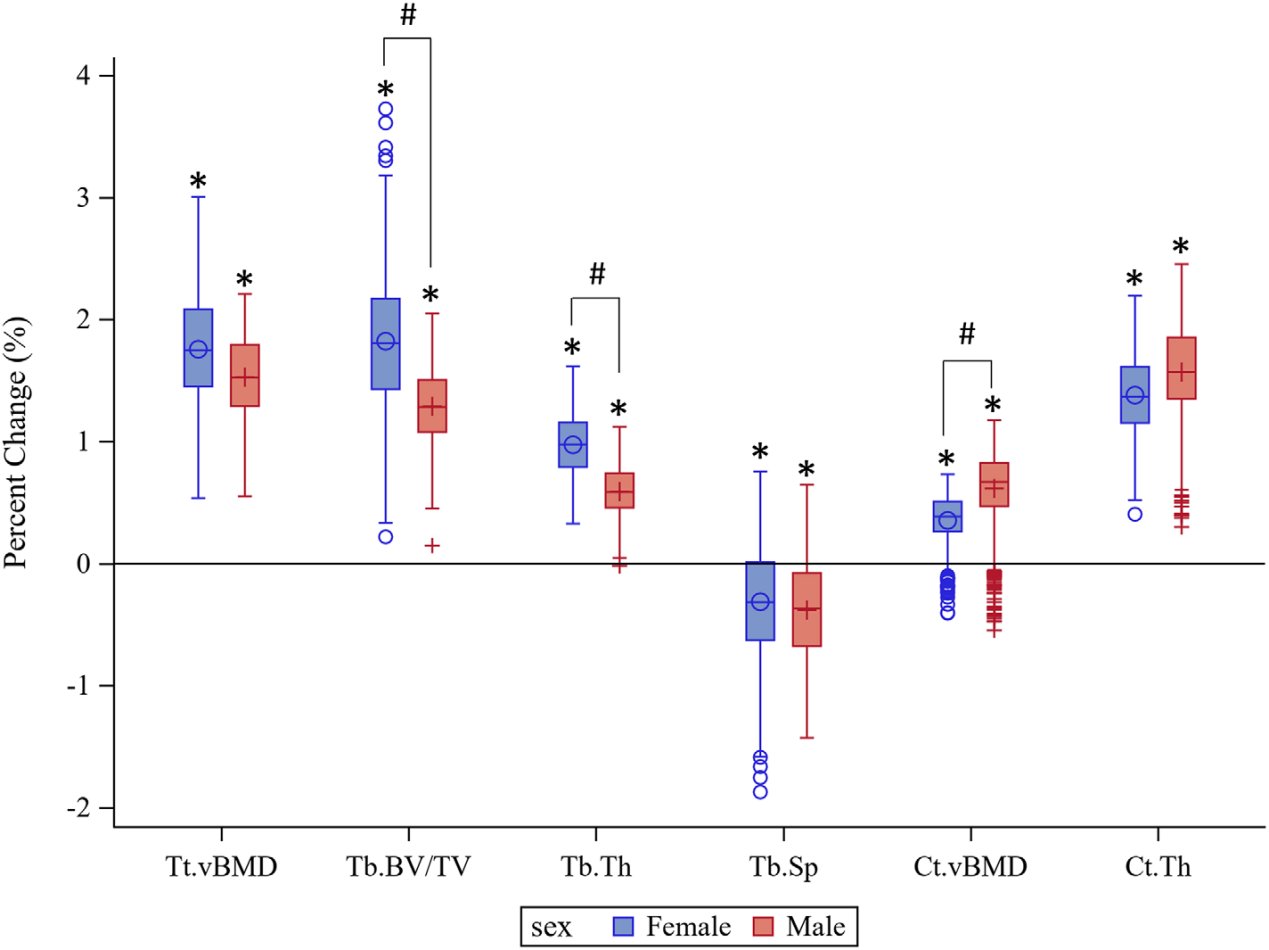
Boxplots of percent change from baseline in bone density and microstructure at the distal tibial metaphysis following 8 weeks of basic combat training in females (blue, O symbol) and males (red, + symbol). Results are adjusted for race, height, weight, tobacco use, and prior physical activity. *Significant difference from baseline (*p* < 0.01). #Significant difference between sexes (*p* ≤ 0.05). Ct.BMD = cortical volumetric bone mineral density; Ct.Th = cortical thickness; Tb.BV/TV = trabecular bone volume/total volume; Tb.Sp = trabecular separation; Tb.Th = trabecular thickness; Tt.BMD = total volumetric bone mineral density.

Eight weeks of BCT led to significant changes in bone density and microarchitecture at the distal tibia. Specifically, after adjusting for age, height, weight, race, prior physical activity, and tobacco use, both males and females showed increases in Tt.BMD, Tb.BMD, Tb.BV/TV, Tb.Th, Tb.N, Ct.BMD, and Ct.Th, and decreases in Tb.Sp (Fig. 2 and Table S3). Increases in Tb.BMD, Tb.BV/TV, and Tb.Th were significantly greater in females than males. In contrast, increases in Ct.BMD were significantly lower in females compared to males. We observed similar patterns by sex when examining absolute changes in bone parameters rather than percent changes (Table S4).

### Changes in bone density and microstructure during BCT: influence of race

**Fig. 3:**
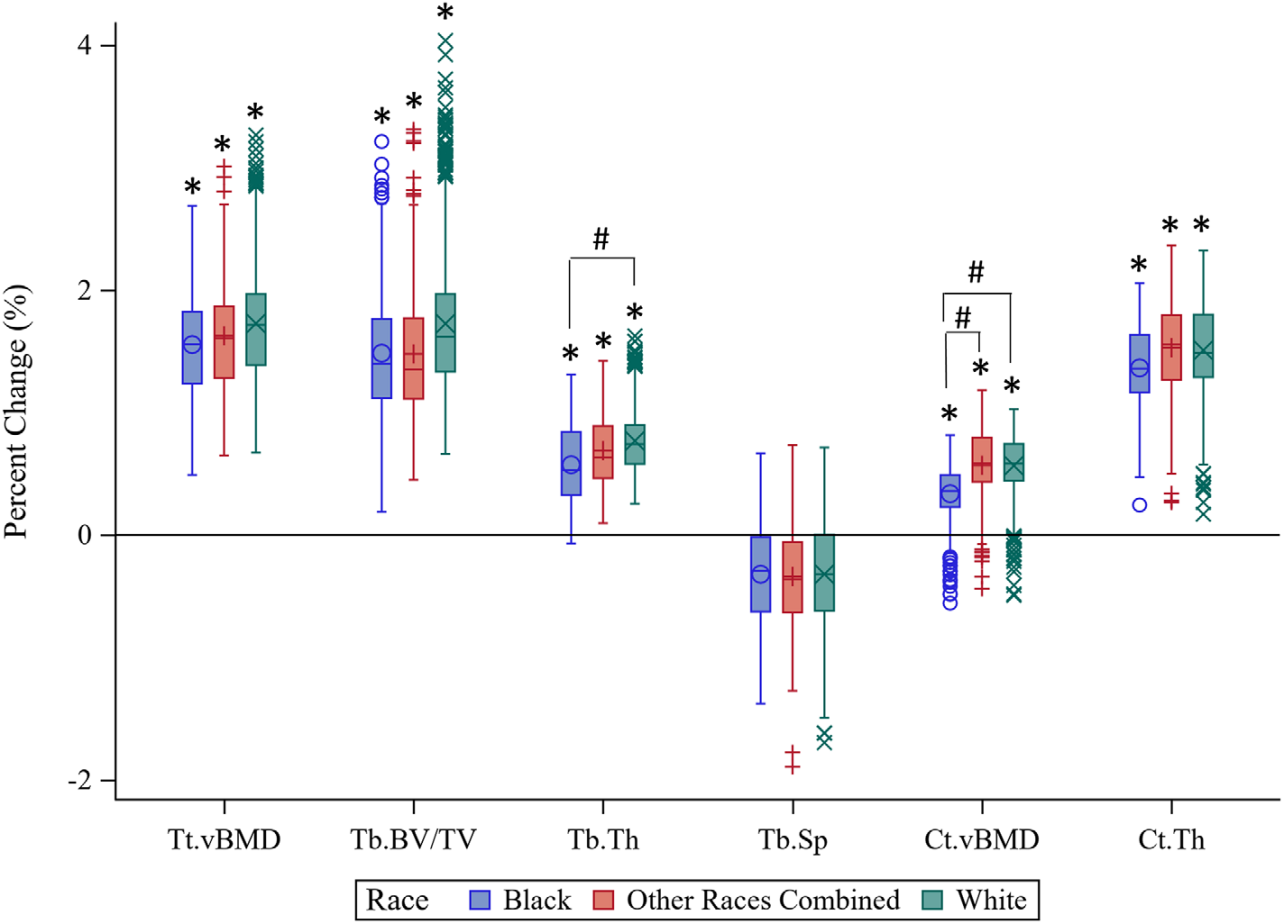
Boxplots of percent change from baseline in bone density and microstructure at the distal tibial metaphysis following 8 weeks of basic combat training in black trainees (blue, O symbol), trainees of other races combined (red, + symbol), and white trainees (green, X symbol). Results are adjusted for sex, height, weight, tobacco use, and prior physical activity. *Significant difference from baseline (*p* < 0.05). #Significant difference from black trainees (*p* < 0.05). Ct.BMD = cortical volumetric bone mineral density; Ct.Th = cortical thickness; Tb.BV/TV = trabecular bone volume/total volume; Tb.Sp = trabecular separation; Tb.Th = trabecular thickness; Tt.BMD = total volumetric bone mineral density.

Black, ORC, and white trainees all exhibited increases in Tt.BMD, Tb.BMD, Tb.BV/TV, Tb.Th, Ct.BMD, and Ct.Th after 8 weeks of BCT (Fig. 3 and Table S5), whereas Tb.Sp. was reduced only in white trainees following BCT, after adjusting for age, height, weight, race, physical activity, and tobacco use. Changes in bone parameters were largely similar among racial groups, with ORC and white trainees exhibiting slightly greater increases in Ct.BMD than black trainees, and white trainees exhibiting greater increases in Tb.Th compared to black trainees. There were no other significant differences by race as a result of BCT. We observed similar patterns by race when examining absolute changes in bone parameters rather than percent changes (Table S6).

### Least significant change in BMD

The LSC for percent change in Tt.BMD is 5.17 g/cm^3^, and 27.7% of participants had an increase in Tt.BMD above the LSC. The LSC for Tb.BMD is 1.01 g/cm^3^, and 75.0% of participants had an increase above the LSC.

## Discussion

This prospective observational study in a large cohort of military trainees allowed us to compare changes in bone density and microstructure during BCT between males and females and among trainees of different races. We observed significant increases in nearly every measured bone property at the distal tibia, in both sexes and across races. Improvements in trabecular bone outcomes were slightly, but significantly, greater in females than males, though were largely similar across individuals of different races.

The magnitudes of change in bone properties that we observed are consistent with prior reports of changes in tibial bone properties during initial military training.^(^ ^2^, ^4^, ^5^, ^22^, ^23^ ^)^ For example, we previously reported 1% to 2% increases in bone density and microarchitecture using HR‐pQCT at the distal tibia in females after 8 weeks of BCT.^(^ ^2^ ^)^ Other studies using pQCT or HR‐pQCT have also reported 0.9% to 2.0% increases in trabecular bone density at the tibia after 8 to 14 weeks of basic combat training in male and female trainees.^(^ ^2^, ^4^, ^5^, ^14^, ^24^ ^)^ An extended period of initial military training (44 weeks) led to larger increases in tibial bone outcomes (2%–4%) in female trainees, consistent with the longer duration of training.^(^ ^23^ ^)^ This current report confirms that these magnitude of changes in bone occur in both males and females and across races.

The bone changes that we observed in response to initial military training are indicative of adaptive bone formation that is experienced with exercise.^(^ ^1^ ^)^ The stimulation of bone formation during BCT is expected because the training includes physically rigorous activities such as resistance exercises, running, hand‐to‐hand combat, and road marches with load carriage.^(^ ^17^, ^25^, ^26^ ^)^ Although the observation of appreciable bone deposition in only 8 weeks is consistent with prior reports,^(^ ^2^, ^4^, ^5^, ^24^ ^)^ these changes were small in magnitude. Because over a quarter and three quarters of participants had increases in Tt.BMD and Tb.BMD, respectively, above the LSC, we can be confident that bone formation occurred, at least in trabecular bone, in the majority of participants during 8 weeks of BCT. The mechanical significance of these increases are unknown. In rats, a small increase in adaptive bone formation at the diaphysis greatly improved the bone's resistance to fatigue loading.^(^ ^7^ ^)^ However, whether the magnitude of bone formation observed in the current study, at the ultradistal tibia, is associated with mechanical benefits that confer protection from stress fracture risk in humans remains to be determined.^(^ ^1^ ^)^ Furthermore, whether bone formation that occurs during BCT is maintained when BCT is complete is also unknown and may depend on how physically rigorous an individual soldier's military occupational specialty is.

Females experienced greater increases in trabecular parameters than males, though males had slightly greater increases in cortical density than females. These sex‐related differences in trabecular and cortical bone responses to military training are consistent with prior studies in female^(^ ^2^ ^)^ and male^(^ ^5^ ^)^ trainees, though no prior study has directly compared sex‐based differences in bone microarchitecture changes during BCT. The difference in the trabecular responses to military training by sex may be partially explained by differences in bone properties at the start of BCT. At the beginning of BCT, females in our study generally had less favorable trabecular and cortical bone properties than males, consistent with prior reports.^(^ ^11^, ^14^, ^27^ ^)^ Worse bone density and structure at military entry could result in greater relative mechanical stimuli in females than in males during military training, and thus, a relatively larger bone anabolic response. Whether the bone formation responses to military training can help compensate for less favorable skeletal phenotypes at the start of BCT,^(^ ^28^ ^)^ and consequently reduce the risk of stress fractures in those able to adapt, remains to be seen.

In contrast to trabecular bone, females had relatively smaller increases than males in cortical bone density. This could be due to sex‐based differences in intracortical remodeling, a process that occurs concurrently with adaptive bone formation in response to heightened mechanical loading.^(^ ^29^, ^30^, ^31^ ^)^ Intracortical remodeling can help replace fatigue‐damaged bone but can also lead to temporary declines in cortical density due to the bone resorption phase preceding the bone formation phase within each remodeling unit. Although the removal of fatigue‐damaged bone is essential to the repair process, this removal transiently increases cortical porosity and reduces cortical bone density, which may contributes to stress fracture pathophysiology.^(^ ^31^ ^)^ However, the physiological bases for this phenomenon, whether it occurs to a greater extent in females, and whether it contributes to the higher rate of stress fractures in females compared to males remains to be determined.

In addition to differences in stress fracture risk in soldiers by sex, there are also differences in stress fracture risk by race and ethnicity.^(^ ^8^ ^)^ Variations in bone properties at the start of BCT and in how bone changes during training among racial groups may both contribute to stress fracture risk. In this study, black trainees generally had more favorable bone density and microstructural properties than ORC and white trainees at the start of BCT. These observed differences in baseline bone values by race are consistent with other studies conducted in civilian populations.^(^ ^12^, ^13^, ^32^ ^)^ Despite differences in bone properties at military entry, in general, BCT‐induced changes in tibial bone density and microstructure differed little by race. These results suggest that differences in risk of stress fracture by race may be largely due to differences in baseline bone properties rather than changes that occur during military training.

There were several strengths of the study, including the longitudinal study design, enrollment of a large, diverse sample of males and females, and use of noninvasive imaging with high resolution and excellent precision, enabling measurement of small changes in bone properties during a relatively brief period of time. This study also had several limitations including the lack of a control group not exposed to the multiple stressors of initial military training. However, in studies conducted in the military training environment it is impractical to include a control group that does not undergo training.^(^ ^2^, ^4^, ^5^, ^23^, ^24^ ^)^ Another limitation is that the study sample size was not robust enough to evaluate bone change during BCT in more granular race and ethnic groups, which limited the analysis to the categories of black, ORC, and white. This approach does not reflect the heterogeneous demography of the U.S. Army.^(^ ^8^ ^)^ The current study did not examine the risk of stress fracture relative to baseline bone properties or changes in bone properties during BCT, but will be a focus of future analyses from this study upon completion of data collection.^(^ ^16^ ^)^ Due to practical limitations of analyzing a large number of pre‐BCT and post‐BCT HR‐pQCT scans, two‐dimensional (2D) registration techniques were used to match baseline and follow‐up scans by bone cross‐sectional area, rather than 3D registration techniques, and therefore, the analyses were not able to account for angular differences between images and potential changes in bone size.^(^ ^33^ ^)^ However, Kemp and colleagues^(^ ^34^ ^)^ reported no advantages of 3D versus 2D registration for longitudinal analyses of the tibia. Finally, changes reflective of bone formation at the ultradistal tibia may not confer advantages in bone properties at other locations in the skeleton that are at risk for stress fractures.

In conclusion, in this prospective observational study in a multiracial cohort of males and females undergoing physically demanding initial military training, we observed changes in trabecular and cortical density and macroarchitecture and microarchitecture at the distal tibia that are indicative of anabolic bone formation in both sexes and across races. The skeletal anabolic response to this rigorous training differed modestly by sex, a finding that may be partially explained by differences in bone properties existing at the start of military training. Whether this bone formation during initial military training confers protection from stress fracture risk has yet to be determined.

## Author Contributions

**Julie M. Hughes:** Conceptualization; data curation; funding acquisition; investigation; methodology; resources; supervision; writing – original draft; writing – review and editing. **Kathryn M. Taylor:** Conceptualization; data curation; formal analysis; methodology; supervision; writing – original draft; writing – review and editing. **Katelyn I. Guerriere:** Conceptualization; data curation; methodology; project administration; resources; software; writing – original draft; writing – review and editing. **Nathaniel I. Smith:** Data curation; resources; writing – original draft; writing – review and editing. **Jeffery S. Staab:** Data curation; writing – original draft; writing – review and editing. **Leila A. Walker:** Conceptualization; data curation; project administration; resources; supervision; writing – original draft; writing – review and editing. **Janet E. Staab:** Data curation; project administration; resources; supervision; writing – original draft; writing – review and editing. **Paul M. Bartlett:** Data curation; resources; software; writing – original draft; writing – review and editing. **Barry A. Spiering:** Conceptualization; data curation; funding acquisition; investigation; supervision; writing – original draft; writing – review and editing. **Vy T. Nguyen:** Data curation; formal analysis; methodology; writing – review and editing. **Susan P. Proctor:** Conceptualization; data curation; funding acquisition; methodology; project administration; resources; supervision; writing – original draft; writing – review and editing. **Stephen A. Foulis:** Conceptualization; data curation; funding acquisition; investigation; project administration; resources; supervision; visualization; writing – original draft; writing – review and editing. **Mary L. Bouxsein:** Conceptualization; methodology; project administration; resources; supervision; visualization; writing – original draft; writing – review and editing. **Kristin L. Popp:** Conceptualization; data curation; methodology; resources; writing – original draft; writing – review and editing.

## Conflict Of Interest

The authors have nothing to disclose.

### Peer Review

The peer review history for this article is available at https://publons.com/publon/10.1002/jbm4.10719.

## Supporting information


**Table S1.** Unadjusted pre‐Basic Combat Training values for bone parameters in males and females.
**Table S2.** Unadjusted pre‐Basic Combat Training values for bone parameters in trainees of Black, Other Races Combined (ORC) and White race.
**Table S3.** Percent change in bone parameters from pre‐ to post‐Basic Combat Training in males and females adjusted for age, height, weight, race, physical activity, and tobacco use.
**Table S4.** Absolute change in bone parameters from pre‐ to post‐Basic Combat Training in males and females adjusted for age, height, weight, race, physical activity, and tobacco use.
**Table S5.** Percent change in bone parameters from pre‐ to post‐Basic Combat Training in trainees of Black, Other Races Combined (ORC), or White race adjusted for age, height, weight, sex, physical activity, and tobacco use.
**Table S6.** Absolute change in bone parameters from pre‐ to post‐Basic Combat Training in trainees of Black, Other Races Combined (ORC), or White race adjusted for age, height, weight, sex, physical activity, and tobacco use.

## Data Availability

The data that support the findings of this study are available from the corresponding author upon reasonable request.
