# Supplementary material for: Changes in Distal Tibial Microarchitecture During Eight Weeks of U.S. Army Basic Combat Training Differ by Sex and Race
Source: JBMR Plus. 2023 Mar 2;7(4):e10719. doi: 10.1002/jbm4.10719 (PMC10097637; doi:10.1002/jbm4.10719)
Supplement: Supplementary file 1 — Table S1. Unadjusted pre‐Basic Combat Training values for bone parameters in males and females. Table S2. Unadjusted pre‐Basic Combat Training values for bone parameters in trainees of Black, Other Races Combined (ORC) and White race. Table S3. Percent change in bone parameters from pre‐ to post‐Basic Combat Training in males and females adjusted for age, height, weight, race, physical activity, and tobacco use. Table S4. Absolute change in bone parameters from pre‐ to post‐Basic Combat Training in males and females adjusted for age, height, weight, race, physical activity, and tobacco use. Table S5. Percent change in bone parameters from pre‐ to post‐Basic Combat Training in trainees of Black, Other Races Combined (ORC), or White race adjusted for age, height, weight, sex, physical activity, and tobacco use. Table S6. Absolute change in bone parameters from pre‐ to post‐Basic Combat Training in trainees of Black, Other Races Combined (ORC), or White race adjusted for age, height, weight, sex, physical activity, and tobacco use. [file JBM4-7-e10719-s001.docx]

**Supplemental Table 1.** Unadjusted pre-Basic Combat Training values for bone parameters in males and females.

|  | Males (n=1053) | Females (n=552) | Males vs. Females | |
| --- | --- | --- | --- | --- |
| Variable | Mean ± SD | Mean ± SD | Mean difference (95% CI) | p |
| Tt.BMD (mgHA/cm^3^) | 274 ± 43 | 254 ± 37 | 20 (16, 24) | <0.001 |
| Tb.BMD (mgHA/cm^3^) | 222 ± 36 | 200 ± 30 | 22 (19, 26) | <0.001 |
| Tb.BV/TV (%) | 32.8 ± 5.3 | 29.4 ± 4.6 | 3.4 (2.9, 4.0) | <0.001 |
| Tb.Th (mm) | 0.246 ± 0.019 | 0.232 ± 0.017 | 0.014 (0.012,0.016) | <0.001 |
| Tb.N (1/mm) | 1.80 ± 0.23 | 1.72 ± 0.20 | 0.07 (0.05,0.10) | <0.001 |
| Tb.Sp (mm) | 0.515 ± 0.078 | 0.539 ± 0.072 | -0.024 (-0.032, -0.016) | <0.001 |
| Ct.BMD (mgHA/cm^3^) | 795 ± 43 | 825 ± 47 | -30 (-35, -26) | <0.001 |
| Ct.Ar (mm^2^) | 108 ± 24 | 82 ± 16 | 26 (24, 28) | <0.001 |
| Ct.Th (mm) | 0.920 ± 0.225 | 0.781 ± 0.175 | 0.139 (0.118, 0.161) | <0.001 |

Tt.BMD=total volumetric bone mineral density; Tb.BMD=trabecular volumetric bone mineral density; Tb.BV/TV=trabecular bone volume/total volume; Tb.Th=trabecular thickness; Tb.N=trabecular number; Tb.Sp=trabecular separation; Ct.BMD=cortical volumetric bone mineral density; Ct.Ar=cortical area; Ct.Th=cortical thickness.

**Supplemental Table 2.** Unadjusted pre-Basic Combat Training values for bone parameters in trainees of Black, Other Races Combined (ORC) and White race.

|  | Black  (n=398) | ORC  (n=306) | White  (n=865) |  | Black vs. ORC | | Black vs. White | | ORC vs. White | |
| --- | --- | --- | --- | --- | --- | --- | --- | --- | --- | --- |
| Variable | Mean ± SD | Mean ± SD | Mean ± SD |  | Mean difference (95% CI) | p | Mean difference (95% CI) | p | Mean difference (95% CI) | p |
| Tt.BMD (mgHA/cm^3^) | 283 ± 44 | 268 ± 40 | 259 ± 40 |  | 15 (9, 21) | <0.001 | 24 (19, 29) | <0.001 | 9 (4, 15) | <0.001 |
| Tb.BMD (mgHA/cm^3^) | 222 ± 37 | 214 ± 34 | 210 ± 35 |  | 8 (3, 13) | 0.004 | 11 (7, 16) | <0.001 | 4 (-1, 8) | 0.118 |
| Tb.BV/TV (%) | 32.9 ± 5.5 | 31.6 ± 5.0 | 31.0 ± 5.2 |  | 1.3 (0.5, 2.1) | 0.002 | 1.9 (1.3, 2.5) | <0.001 | 0.9 (-0.1, 1.3) | 0.079 |
| Tb.Th (mm) | 0.248 ± 0.021 | 0.242 ± 0.020 | 0.237 ± 0.018 |  | 0.006 (0.003, 0.009) | <0.001 | 0.010 (0.008, 0.013) | <0.001 | 0.004 (0.002, 0.007) | 0.002 |
| Tb.N (1/mm) | 1.73 ± 0.22 | 1.75 ± 0.21 | 1.80 ± 0.22 |  | -0.02 (-0.06, 0.01) | 0.137 | -0.07 (-0.10, -0.05) | <0.001 | -0.05 (-0.08, -0.02) | <0.001 |
| Tb.Sp (mm) | 0.535 ± 0.084 | 0.530 ± 0.073 | 0.516 ± 0.074 |  | 0.004 (-0.007, 0.016) | 0.455 | 0.019 (0.010, 0.028) | <0.001 | 0.015 (0.005, 0.024) | 0.004 |
| Ct.BMD (mgHA/cm^3^) | 834 ± 42 | 806 ± 47 | 792 ± 43 |  | 28 (21, 34) | <0.001 | 43 (37, 48) | <0.001 | 15 (9, 20) | <0.001 |
| Ct.Ar (mm^2^) | 109 ± 28 | 99 ± 25 | 95 ± 22 |  | 10 (6, 14) | <0.001 | 14 (11, 17) | <0.001 | 4 (0, 7) | 0.025 |
| Ct.Th (mm) | 0.978 ± 0.247 | 0.881 ± 0.202 | 0.820 ± 0.189 |  | 0.097 (0.065, 0.128) | <0.001 | 0.157 (0.133, 0.183) | <0.001 | 0.061 (0.033, 0.088) | <0.001 |

Tt.BMD=total volumetric bone mineral density; Tb.BMD=trabecular volumetric bone mineral density; Tb.BV/TV=trabecular bone volume/total volume; Tb.Th=trabecular thickness; Tb.N=trabecular number; Tb.Sp=trabecular separation; Ct.BMD=cortical volumetric bone mineral density; Ct.Ar=cortical area; Ct.Th=cortical thickness.

**Supplemental Table 3.** Percent change in bone parameters from pre- to post-Basic Combat Training in males and females adjusted for age, height, weight, race, physical activity, and tobacco use.

|  | Males (n=1053) |  | Females (n=552) |  | Males vs. Females |
| --- | --- | --- | --- | --- | --- |
| Variable | Mean % change (95% CI) | p vs baseline | Mean % change (95% CI) | p vs baseline | p |
| Tt.BMD (mgHA/cm^3^) | 1.53 (1.38, 1.68) | <0.001 | 1.76 (1.54, 2.00) | <0.001 | 0.129 |
| Tb.BMD (mgHA/cm^3^) | 1.40 (1.21, 1.58) | <0.001 | 1.87 (1.60, 2.14) | <0.001 | 0.009 |
| Tb.BV/TV (%) | 1.28 (1.10, 1.46) | <0.001 | 1.82 (1.56, 2.08) | <0.001 | 0.002 |
| Tb.Th (mm) | 0.58 (0.46, 0.70) | <0.001 | 0.87 (0.69, 1.04) | <0.001 | 0.016 |
| Tb.N (1/mm) | 0.46 (0.10, 0.82) | <0.001 | 0.20 (-0.33, 0.72) | <0.001 | 0.462 |
| Tb.Sp (mm) | -0.37 (-0.65, -0.09) | <0.001 | -0.31 (-0.72, 0.10) | <0.001 | 0.830 |
| Ct.BMD (mgHA/cm^3^) | 0.61 (0.54, 0.67) | <0.001 | 0.35 (0.25, 0.44) | <0.001 | <0.001 |
| Ct.Th (mm) | 1.56 (1.34, 1.77) | <0.001 | 1.37 (1.05, 1.68) | <0.001 | 0.365 |

Tt.BMD=total volumetric bone mineral density; Tb.BMD=trabecular volumetric bone mineral density; Tb.BV/TV=trabecular bone volume/total volume; Tb.Th=trabecular thickness; Tb.N=trabecular number; Tb.Sp=trabecular separation; Ct.BMD=cortical volumetric bone mineral density; Ct.Th=cortical thickness.

**Supplemental Table 4.** Absolute change in bone parameters from pre- to post-Basic Combat Training in males and females adjusted for age, height, weight, race, physical activity, and tobacco use.

|  | Males (n=1053) |  | Females (n=552) |  | Males vs. Females |
| --- | --- | --- | --- | --- | --- |
| Variable | Mean change (95% CI) | p vs baseline | Mean change (95% CI) | p vs baseline | p |
| Tt.BMD (mgHA/cm^3^) | 3.87 (3.55, 4.19) | <0.001 | 4.15 (3.68, 4.63) | <0.001 | 0.373 |
| Tb.BMD (mgHA/cm^3^) | 2.74 (2.44, 3.05) | <0.001 | 3.41 (2.96, 3.86) | <0.001 | 0.029 |
| Tb.BV/TV (%) | 0.37 (0.33, 0.42) | <0.001 | 0.48 (0.41, 0.55) | <0.001 | 0.014 |
| Tb.Th (mm) | 0.001 (0.001, 0.002) | <0.001 | 0.002 (0.002, 0.002) | <0.001 | 0.491 |
| Tb.N (1/mm) | 0.006 (-0.000, 0.012) | 0.066 | 0.002 (-0.007, 0.012) | 0.619 | 0.566 |
| Tb.Sp (mm) | -0.002 (-0.004, -0.001) | 0.001 | -0.002 (-0.004, 0.000) | 0.085 | 0.733 |
| Ct.BMD (mgHA/cm^3^) | 4.69 (4.19, 5.19) | <0.001 | 2.73 (2.00, 3.46) | <0.001 | <0.001 |
| Ct.Th (mm) | 0.013 (0.011, 0.015) | <0.001 | 0.010 (0.007, 0.013) | <0.001 | 0.151 |

Tt.BMD=total volumetric bone mineral density; Tb.BMD=trabecular volumetric bone mineral density; Tb.BV/TV=trabecular bone volume/total volume; Tb.Th=trabecular thickness; Tb.N=trabecular number; Tb.Sp=trabecular separation; Ct.BMD=cortical volumetric bone mineral density; Ct.Th=cortical thickness.

**Supplemental Table 5.** Percent change in bone parameters from pre- to post-Basic Combat Training in trainees of Black, Other Races Combined (ORC), or White race adjusted for age, height, weight, sex, physical activity, and tobacco use.

|  | Black  (n=398) | | ORC  (n=306) | | White  (n=865) | |  | Black vs. ORC | Black vs. White | ORC vs. White |
| --- | --- | --- | --- | --- | --- | --- | --- | --- | --- | --- |
| Variable | Mean  (95% CI) | p vs baseline | Mean  (95% CI) | p vs baseline | Mean  (95% CI) | p vs baseline |  | p | p | p |
| Tt.BMD (mgHA/cm^3^) | 1.55 (1.35, 1.74) | <0.001 | 1.63 (1.39, 1.87) | <0.001 | 1.76 (1.61, 1.90) | <0.001 |  | 0.588 | 0.085 | 0.348 |
| Tb.BMD (mgHA/cm^3^) | 1.60 (1.36, 1.84) | <0.001 | 1.55 (1.26, 1.84) | <0.001 | 1.76 (1.58, 1.94) | <0.001 |  | 0.779 | 0.273 | 0.195 |
| Tb.BV/TV (%) | 1.46 (1.13, 1.69) | <0.001 | 1.46 (1.18, 1.74) | <0.001 | 1.73 (1.55, 1.90) | <0.001 |  | 0.993 | 0.066 | 0.094 |
| Tb.Th (mm) | 0.61 (0.46, 0.77) | <0.001 | 0.74 (0.55, 0.93) | <0.001 | 0.82 (0.70, 0.94) | <0.001 |  | 0.301 | 0.033 | 0.443 |
| Tb.N (1/mm) | 0.39 (-0.08, 0.86) | 0.103 | 0.32 (-0.24, 0.87) | 0.268 | 0.28 (-0.07, 0.63) | 0.112 |  | 0.836 | 0.708 | 0.915 |
| Tb.Sp (mm) | -0.33 (-0.70, 0.04) | 0.077 | -0.35 (-0.79, 0.08) | 0.114 | -0.34 (-0.61, -0.06) | 0.015 |  | 0.939 | 0.980 | 0.949 |
| Ct.BMD (mgHA/cm^3^) | 0.32 (0.23, 0.40) | <0.001 | 0.56 (0.46, 0.66) | <0.001 | 0.55 (0.49, 0.62) | <0.001 |  | <0.001 | <0.001 | 0.918 |
| Ct.Th (mm) | 1.35 (1.06, 1.63) | <0.001 | 1.51 (1.18, 1.85) | <0.001 | 1.53 (1.32, 1.74) | <0.001 |  | 0.448 | 0.301 | 0.929 |

Tt.BMD=total volumetric bone mineral density; Tb.BMD=trabecular volumetric bone mineral density; Tb.BV/TV=trabecular bone volume/total volume; Tb.Th=trabecular thickness; Tb.N=trabecular number; Tb.Sp=trabecular separation; Ct.BMD=cortical volumetric bone mineral density; Ct.Th=cortical thickness.

**Supplemental Table 6.** Absolute change in bone parameters from pre- to post-Basic Combat Training in trainees of Black, Other Races Combined (ORC), or White race adjusted for age, height, weight, sex, physical activity, and tobacco use.

|  | Black  (n=398) | | ORC  (n=306) | | White  (n=865) | |  | Black vs. ORC | Black vs. White | ORC vs. White |
| --- | --- | --- | --- | --- | --- | --- | --- | --- | --- | --- |
| Variable | Mean  (95% CI) | p vs baseline | Mean  (95% CI) | p vs baseline | Mean  (95% CI) | p vs baseline |  | p | p | p |
| Tt.BMD (mgHA/cm^3^) | 3.96 (3.54, 4.38) | <0.001 | 3.98 (3.48, 4.49) | <0.001 | 4.10 (3.79, 4.41) | <0.001 |  | 0.939 | 0.576 | 0.678 |
| Tb.BMD (mgHA/cm^3^) | 3.09 (2.69, 3.48) | <0.001 | 2.95 (2.47, 3.42) | <0.001 | 3.19 (2.89, 3.49) | <0.001 |  | 0.654 | 0.670 | 0.378 |
| Tb.BV/TV (%) | 0.41 (0.36, 0.47) | 0.004 | 0.40 (0.33, 0.47) | 0.004 | 0.46 (0.42, 0.50) | 0.005 |  | 0.820 | 0.181 | 0.143 |
| Tb.Th (mm) | 0.001 (0.001, 0.002) | 0.001 | 0.002 (0.001, 0.002) | 0.002 | 0.002 (0.002, 0.002) | 0.002 |  | 0.337 | 0.566 | 0.448 |
| Tb.N (1/mm) | 0.006 (-0.003, 0.014) | 0.006 | 0.004 (-0.006, 0.014) | 0.005 | 0.003 (-0.003, 0.009) | 0.003 |  | 0.811 | 0.53 | 0.807 |
| Tb.Sp (mm) | -0.002 (-0.004, -0.000) | 0.045 | -0.002 (-0.004, 0.000) | 0.064 | -0.002 (-0.004, -0.001) | 0.002 |  | 0.892 | 0.778 | 0.917 |
| Ct.BMD (mgHA/cm^3^) | 2.57 (1.92, 3.23) | <0.001 | 4.31 (3.54, 5.09) | <0.001 | 4.24 (3.76, 4.73) | <0.001 |  | 0.001 | <0.001 | 0.878 |
| Ct.Th (mm) | 0.012 (0.009, 0.014) | <0.001 | 0.011 (0.008, 0.014) | <0.001 | 0.011 (0.010, 0.013) | <0.001 |  | 0.892 | 0.851 | 0.986 |

Tt.BMD=total volumetric bone mineral density; Tb.BMD=trabecular volumetric bone mineral density; Tb.BV/TV=trabecular bone volume/total volume; Tb.Th=trabecular thickness; Tb.N=trabecular number; Tb.Sp=trabecular separation; Ct.BMD=cortical volumetric bone mineral density; Ct.Th=cortical thickness.
